# Unveiling the Clinical Diversity in Nontuberculous Mycobacteria (NTM) Infections: A Comprehensive Review

**DOI:** 10.7759/cureus.48270

**Published:** 2023-11-04

**Authors:** Jay Bhanushali, Ulhas Jadhav, Babaji Ghewade, Pankaj Wagh

**Affiliations:** 1 Respiratory Medicine, Jawaharlal Nehru Medical College, Datta Meghe Institute of Higher Education and Research, Wardha, IND

**Keywords:** research trends, prevention, treatment, diagnostics, clinical diversity, epidemiology, ntm infections, nontuberculous mycobacteria

## Abstract

Once considered rare, nontuberculous mycobacterial (NTM) infections have garnered increasing attention in recent years. This comprehensive review provides insights into the epidemiology, clinical diversity, diagnostic methods, treatment strategies, prevention, and emerging research trends in NTM infections. Key findings reveal the global prevalence of NTM infections, their diverse clinical presentations affecting respiratory and extra-pulmonary systems, and the diagnostic challenges addressed by advances in microbiological, radiological, and immunological methods. Treatment complexities, especially drug resistance and patient adherence, are discussed, along with the vulnerability of special populations. The importance of early detection and management is underscored. Prospects in NTM research, including genomics, diagnostics, drug development, and multidisciplinary approaches, promise to enhance our understanding and treatment of these infections. This review encapsulates the multifaceted nature of NTM infections, offering a valuable resource for clinicians, researchers, and public health professionals.

## Introduction and background

Nontuberculous mycobacteria (NTM) infections, caused by diverse mycobacterial species other than Mycobacterium tuberculosis and Mycobacterium leprae, have gained increasing recognition in recent years. Historically considered opportunistic pathogens primarily affecting individuals with compromised immune systems, NTM infections have emerged as a significant clinical concern among both immunocompromised and immunocompetent populations. These infections are characterized by their ability to affect various organ systems, leading to a broad spectrum of clinical manifestations [1-3].

NTM species are ubiquitous in the environment, thriving in soil, water, and biofilms, which increases the potential for human exposure. The clinical relevance of NTM infections has expanded beyond curiosity in infectious diseases to a substantial public health issue with a considerable economic burden associated with diagnosis and treatment [4]. Understanding and addressing NTM infections is of paramount importance for several reasons. Firstly, the incidence of NTM infections is rising globally, exhibiting considerable clinical diversity. Consequently, healthcare professionals, researchers, and policymakers face the challenge of effectively recognizing, diagnosing, and managing this intricate array of diseases [5]. Secondly, NTM infections can be chronic and debilitating, often requiring prolonged and complex treatment regimens. The consequences of misdiagnosis or delayed treatment can lead to irreversible tissue damage and diminished quality of life for affected individuals [6]. Furthermore, NTM infections can mimic other respiratory diseases, such as tuberculosis, making accurate diagnosis crucial in inpatient care. The increasing prevalence of NTM infections, along with the ongoing challenges of antimicrobial resistance, necessitates a comprehensive review to synthesize current knowledge and promote better clinical practices [7].

The primary purpose of this comprehensive review is to illuminate the inherent clinical diversity within NTM infections. Our objective is to equip healthcare professionals, researchers, and policymakers with a comprehensive understanding of NTM infections, encompassing their epidemiology, clinical manifestations, diagnostic methodologies, management approaches, and emerging trends. In pursuing these objectives, we intend to achieve several key outcomes: Firstly, we seek to facilitate early detection and accurate diagnosis by exploring the array of clinical presentations and diagnostic modalities associated with NTM infections. This, in turn, aims to enhance timely interventions, ultimately improving patient outcomes. Secondly, our review delves into the intricate landscape of treatment strategies, including emerging therapeutic options, to provide healthcare providers with the knowledge necessary to select the most suitable treatments, further optimizing patient care. Thirdly, we address the broader public health implications of NTM infections, recognizing their challenges regarding infection control.

## Review

Epidemiology of NTM infections

Global Prevalence and Incidence

NTM infections have emerged as a global health concern, with steadily increasing prevalence and incidence rates over recent decades. The global prevalence of NTM in adults with non-CF bronchiectasis from 2006 to 2021 was estimated to be approximately 10%, with great variations primarily due to geographical location. Mycobacterium avium complex was the most common subspecies, followed by Mycobacterium simiae and Mycobacterium gordonae [8]. These infections are not confined to specific geographic regions or populations, and they pose significant challenges in quantifying their exact burden due to variations in reporting and underdiagnosis. However, several pivotal aspects underscore the global significance of NTM infections.

Escalating incidence: An alarming trend of rising NTM infection incidence has been documented in multiple countries worldwide. The annual incidence of NTM lung disease increased from 3.13 (95% CI, 2.88-3.40) in 2008 to 4.73 (95% CI, 4.43-5.05) in 2015 per 100,000 person-years. The average rate of yearly change for incidence was +5.2% (95% CI, 4.0-6.4; P < 0.01). This upward trajectory can be attributed, at least in part, to advancements in diagnostic techniques and heightened awareness among healthcare professionals. These improvements have enabled more accurate detection and reporting of NTM infections [9].

Regional disparities: While NTM infections have a worldwide presence, pronounced regional variations exist in their prevalence. The pooled estimate for the point prevalence of NTM infection was 7.9% (95% CI 5.1-12.0%). Certain regions, including North America, Japan, and specific parts of Europe, have reported higher rates of NTM infections, whereas others exhibit a comparatively lower incidence. These disparities may result from a complex interplay of genetic, environmental, and healthcare system factors [10].

Geographical clustering: Within regions with a high incidence of NTM infections, one can observe a geographical clustering of particular NTM species. This phenomenon suggests that environmental factors, such as climate conditions and water sources, may substantially influence the distribution and prevalence of NTM infections. Understanding these environmental determinants is crucial for effective prevention and management strategies [11].

Geographical Distribution

Environmental reservoirs: NTM species find their niches in diverse environmental reservoirs, including natural water sources like rivers, lakes, and soil. Additionally, human-made biofilms within plumbing systems serve as thriving grounds for these bacteria. Regions abundant in such reservoirs are more likely to experience higher rates of NTM infections. These bacteria can be inadvertently introduced into water supplies, leading to potential exposure during everyday activities like showering or drinking tap water [12].

Climate influence: Climate conditions play a significant role in shaping the prevalence of NTM infections. Temperature and humidity are particularly influential factors. Some NTM species are more prevalent in warmer, humid climates, while others thrive in more relaxed environments. For instance, the warmer and more humid climates of tropical and subtropical regions may favor the growth of Mycobacterium avium complex (MAC) and Mycobacterium abscessus. Conversely, Mycobacterium kansasii, which prefers more relaxed conditions, is more commonly encountered in temperate regions [13].

Species-specific distribution: Different NTM species exhibit distinct global distributions. This variation can be attributed to various factors, including environmental preferences and regional reservoirs. For example, Mycobacterium avium complex (MAC) is frequently isolated in North America, particularly in areas with substantial environmental water sources. Conversely, Mycobacterium kansasii is more commonly found in Europe, where climatic conditions and environmental reservoirs may be more conducive to its growth [14].

Risk Factors for NTM Infections

Immunosuppression: Individuals with compromised immune systems, such as those living with HIV/AIDS, organ transplant recipients, or individuals on immunosuppressive medications, face a heightened risk of NTM infections. In these populations, NTM infections can manifest as disseminated disease, where the bacteria spread throughout the body, causing severe and widespread illness. The weakened immune response makes it more challenging for the body to control NTM bacteria [15].

Chronic lung disease: People with pre-existing lung conditions, such as bronchiectasis, chronic obstructive pulmonary disease (COPD), or cystic fibrosis, are particularly susceptible to pulmonary NTM infections, including nontuberculous mycobacterial lung disease (NTMLD). These underlying lung conditions create an environment where NTM bacteria can thrive, leading to chronic and potentially severe respiratory infections [16].

Age: Elderly individuals face an elevated risk of NTM infections, possibly due to age-related changes in the immune system and a higher likelihood of having underlying medical conditions. Age-related immune system decline can diminish the body's ability to defend against NTM bacteria, making older individuals more susceptible to these infections [17].

Environmental exposure: Engaging in activities that involve exposure to soil or water, such as gardening, farming, or specific occupational settings (e.g., construction work near water sources), may increase the risk of NTM infections. NTM bacteria can be inhaled or come into contact with the skin and soft tissues during these activities. Inhalation of NTM-contaminated aerosols is a common route for pulmonary infections, particularly among individuals with pre-existing lung conditions [18].

Smoking: An increased risk of NTM lung infections has been associated with it, especially in individuals with pre-existing lung conditions. Smoking damages the respiratory epithelium and impairs the lung's natural defense mechanisms, creating an environment conducive to NTM colonization and infection [19].

Genetic Factors: Although not fully understood, some genetic predispositions have been linked to susceptibility to NTM infections. Variations in immune-related genes may play a role in determining an individual's ability to mount an effective immune response against NTM bacteria. Research in this area continues to uncover genetic factors contributing to NTM susceptibility [20].

Classification and taxonomy of NTM

Mycobacterium Species Implicated in NTM Infections

Nontuberculous mycobacteria (NTM) infections are caused by diverse mycobacterial species, each with distinct characteristics and clinical implications. Recognizing this diversity is crucial for accurate diagnosis and effective treatment. Among the prominent NTM species commonly implicated in human infections, the Mycobacterium avium complex (MAC) stands out. This complex includes Mycobacterium avium and Mycobacterium intracellulare, which have a propensity for causing pulmonary NTM infections, particularly in individuals with preexisting lung conditions [21].

Another noteworthy NTM species is Mycobacterium kansasii, known for its association with NTM lung disease, primarily prevalent in Europe and parts of North America. It can manifest with clinical and radiological features closely resembling tuberculosis, making diagnosis challenging [22]. On the other hand, Mycobacterium abscess is notorious for its multidrug resistance, posing a substantial hurdle to treatment. It can lead to various infections, including skin and soft tissue infections, pulmonary infections, and even disseminated disease, making it a formidable adversary in clinical settings [23].

Mycobacterium chelonae is primarily associated with skin and soft tissue infections, often linked to cosmetic and medical procedures. This species is notorious for its antibiotic resistance, making treatment complex and protracted [24]. In contrast, Mycobacterium xenopi is commonly found in environmental sources, notably tap water. It primarily affects individuals with underlying lung diseases and poses a unique challenge in the context of NTM infections.

Phylogenetic Relationships

Diversity within genera: NTM species are classified within the genus Mycobacterium, which also encompasses the well-known Mycobacterium tuberculosis, responsible for tuberculosis. However, despite their shared genus, NTM species exhibit significant genetic diversity and are distinct from M. tuberculosis. This genetic divergence accounts for the differences in their clinical presentation, virulence, and susceptibility to treatment [25].

16S rRNA gene sequencing: Phylogenetic studies of NTM often rely on genetic markers such as the 16S ribosomal RNA (rRNA) gene to assess relatedness among various NTM species. This gene has become a cornerstone in deciphering the evolutionary relationships within the NTM group. By comparing the sequences of this gene, researchers can discern the genetic distinctions that separate different NTM species and understand their evolutionary branching patterns [26].

Genomic analyses: Recent advancements in genomics have significantly expanded the scope of phylogenetic investigations within the NTM group. The availability of whole-genome sequencing techniques has enabled researchers to conduct more comprehensive and nuanced analyses, revealing the intricate genetic diversity within NTM species. This in-depth genomic data not only aids in discerning phylogenetic relationships but also provides insights into the genes and pathways that contribute to the unique characteristics and virulence of different NTM strains [27].

Clinical Relevance of NTM Species

Pulmonary vs. extra-pulmonary infections: NTM species exhibit a broad spectrum of clinical manifestations. Some are predominantly associated with pulmonary infections, affecting the lungs primarily. In contrast, others are more likely to cause extra-pulmonary infections, manifesting as skin and soft tissue infections or disseminated diseases involving multiple organ systems. Recognizing these differences is crucial for accurate diagnosis and tailored treatment approaches [3].

Species-specific virulence: NTM species vary in their virulence and resistance to antimicrobial agents. For instance, Mycobacterium abscessus is notorious for its high virulence and resistance to many antibiotics, including those typically used to treat NTM infections. This heightened virulence makes M. abscessus infections particularly challenging to manage and necessitates specialized treatment strategies [28].

Geographical variation: The prevalence of specific NTM species can vary significantly by geographic region. This geographical variation can lead to differences in clinical presentations and infection patterns. Healthcare providers must consider regional variations when diagnosing and managing NTM infections. For example, Mycobacterium kansasii is more commonly found in Europe and parts of North America, while Mycobacterium xenopi is often associated with environmental sources like tap water [1].

Antibiotic susceptibility: NTM species differ in their susceptibility to antibiotics. Treatment of NTM infections often relies on a combination of antibiotics tailored to the specific infecting species and its drug susceptibility profile. Understanding the susceptibility profile of the NTM species involved is crucial for selecting the most effective treatment regimen, as using inappropriate antibiotics can lead to treatment failure and the development of antibiotic resistance [1].

Clinical presentation of NTM infections

Respiratory NTM Infections

These infections encompass a diverse range of pulmonary conditions caused by various species of mycobacteria other than Mycobacterium tuberculosis. Among the most notable manifestations are pulmonary NTM infections, which can be particularly challenging to diagnose due to their mimicry of other respiratory diseases. Chronic bronchitis is a common presentation where patients exhibit persistent cough, sputum production, and bronchial inflammation, often resembling chronic obstructive pulmonary disease (COPD). NTM infections can also lead to cavitary lung disease, characterized by the formation of cavities in the lung parenchyma. This can result in symptoms such as hemoptysis (coughing up blood), weight loss, and fatigue. Another subset of individuals may develop fibrocavitary disease, distinguished by cavities surrounded by fibrotic tissue [5].

Additionally, nontuberculous mycobacterial lung disease (NTMLD) encompasses distinct clinical entities. One example is Lady Windermere syndrome, typically observed in middle-aged women. This syndrome is associated with right middle lobe or lingular segmental bronchiectasis and often manifests as a chronic cough with or without sputum production. NTMLD can also lead to fibrodular bronchiectasis, characterized by bronchiectasis (abnormal dilation of bronchial tubes) and the formation of nodules within the lungs, resulting in persistent respiratory symptoms [29].

Clinical symptoms and radiological findings are crucial in diagnosing these respiratory NTM infections. Patients commonly report chronic cough, hemoptysis, fatigue, and unintentional weight loss, which are important clinical indicators. Radiological findings can vary, including bronchiectasis, pulmonary nodules, infiltrates, or cavities. High-resolution computed tomography (HRCT) is particularly valuable for diagnosis, as it can provide detailed images of the lung structures, aiding in identifying characteristic abnormalities associated with NTM infections. Early and accurate diagnosis is essential for implementing appropriate treatment strategies, often involving a combination of antibiotics and, in some cases, surgical intervention to manage the complex spectrum of respiratory NTM infections effectively [30].

Extra-pulmonary NTM Infections

Skin and soft tissue infections: NTM can give rise to localized cutaneous infections, resulting in nodules, abscesses, or ulcers on the skin. These infections may require either surgical or medical interventions for proper management. Moreover, postsurgical NTM infections can occur, particularly following cosmetic or plastic surgery procedures, highlighting the importance of proper infection control measures in healthcare settings [31].

Disseminated infections: In some cases, NTM infections can disseminate from the initial site of infection to other organs, leading to systemic symptoms like fever, fatigue, and unintentional weight loss. These disseminated NTM infections are more frequently encountered in individuals with compromised immune systems, such as those with HIV/AIDS or organ transplant recipients [32].

Bone and joint infections: NTM-related osteomyelitis and joint infections can manifest with localized pain, swelling, and reduced joint mobility. Diagnosis typically involves imaging studies, such as X-rays or MRIs, along with the culture of affected tissues [33].

Lymphadenitis: Cervical lymphadenitis commonly presents NTM infections, primarily affecting children. Painless, enlarged lymph nodes in the neck characterize it. Diagnosis often necessitates a lymph node biopsy and culture to identify the specific NTM species involved [34].

Ocular NTM infections: NTM can cause eye infections, resulting in redness, pain, blurred vision, and eye discharge. Diagnosis may require ocular swabs, biopsies, and specialized eye examinations by ophthalmologists to determine the precise NTM species and initiate targeted treatment [35].

Other rare manifestations: NTM infections have been sporadically reported in various organs, including the heart, liver, and central nervous system, although such occurrences are less common. These rare manifestations often pose diagnostic challenges and may necessitate specialized approaches, including advanced imaging techniques, biopsies, and culture-based identification, for accurate diagnosis and tailored treatment strategies [5].

Diagnostic methods for NTM infections

Microbiological Methods

Sputum collection: The diagnostic process often involves collecting sputum samples from patients presenting respiratory symptoms. Induced sputum, obtained through inhalation of a hypertonic saline solution, or early morning samples are preferred as they tend to yield a higher concentration of mycobacteria, increasing the chances of detection [36]. Sputum samples are subsequently subjected to culture on specialized NTM agar or broth media. This critical step involves incubating the samples for several weeks at a controlled temperature. NTM colonies, if present, will gradually grow during this incubation period [37].

Species identification: After growth is observed, the colonies are subjected to further analysis for species identification. Traditional biochemical tests can be employed for this purpose, which involves assessing various metabolic activities and characteristics of the isolated bacteria. In recent years, molecular techniques have gained prominence in the identification process. DNA sequencing or PCR-based assays targeting specific NTM genes or regions (such as 16S rRNA or hsp65) are increasingly utilized for their accuracy and specificity in determining the NTM species present in the clinical sample. These molecular methods provide rapid and precise species identification, which is crucial for tailoring effective treatment regimens [38].

Biopsy and Tissue Cultures

Tissue biopsy: In cases where NTM infections manifest as extra-pulmonary conditions, such as skin and soft tissue infections, lymphadenitis, and bone or joint infections, clinicians may perform a tissue biopsy. This involves surgically removing a small piece of affected tissue, such as skin, lymph nodes, or bone [39].

Culture: Tissue samples obtained through biopsies are cultured on appropriate mycobacterial media. The culturing process for tissue samples parallels that of sputum cultures, involving incubation over several weeks. This step is crucial for isolating the NTM and confirming the diagnosis [40].

Species identification: Once growth is observed, similar protocols for species identification are followed, as in sputum cultures. Biochemical tests and molecular techniques, such as DNA sequencing or polymerase chain reaction (PCR), are employed to accurately identify the NTM species involved [41].

Molecular Techniques

Polymerase chain reaction (PCR) assays have become indispensable tools for rapidly and accurately identifying NTM species in clinical specimens. These assays target specific genes or regions of the NTM genome, such as the 16S rRNA or hsp65 genes, which are highly conserved and specific to mycobacteria [42]. PCR offers several advantages, including high sensitivity and specificity and the ability to provide rapid results. It can detect even low levels of NTM DNA in clinical samples, facilitating early diagnosis and timely initiation of treatment. PCR-based assays are precious in cases where traditional culture methods may be slow or challenging due to the slow growth characteristics of NTM [43].

Radiological Imaging

This is an essential component in diagnosing and assessing NTM infections, offering valuable insights into the extent and characteristics of the disease. Among the imaging techniques employed, chest X-rays serve as a preliminary tool that detects noticeable irregularities like nodules, cavities, or infiltrates within the pulmonary region. However, it's crucial to note that while chest X-rays provide a quick initial assessment, they may need more intricate details for a comprehensive evaluation [44].

Stepping up in sophistication and precision, high-resolution computed tomography (HRCT) emerges as a pivotal imaging method for pulmonary NTM infections. HRCT scans delve deeper into the lung parenchyma, affording a more comprehensive view. This advanced imaging technique proves particularly beneficial in identifying characteristic features associated with NTM infections, such as bronchiectasis and nodules. It is crucial for accurately diagnosing and monitoring the disease's progression [45].

Furthermore, when NTM infections extend beyond the pulmonary system into other organs, alternative imaging modalities come into play. For instance, when assessing extra-pulmonary NTM infections, specific imaging studies tailored to the affected organ system become indispensable. In such cases, magnetic resonance imaging (MRI) is a valuable tool for evaluating issues related to joints or the central nervous system (CNS). The choice of imaging modality is guided by the location and nature of the infection, ensuring that clinicians can precisely diagnose and strategize treatment plans for NTM infections across various clinical scenarios [39].

Serological and Immunological Assays

Serological assays are designed to detect specific antibodies produced by the patient's immune system in response to NTM antigens. These tests can help diagnose NTM infections, as the presence of antibodies can indicate exposure to the bacteria. However, it's important to note that serological assays may not always provide definitive results, and there is a risk of cross-reactivity with antibodies produced in response to other mycobacterial infections. Therefore, while serological assays can be informative, they are typically used with other diagnostic methods for a more comprehensive assessment [46].

Immunological assays, such as the interferon-gamma release assay (IGRA), are particularly valuable for identifying exposure to mycobacterial antigens, including those from NTM. IGRA is especially useful in screening individuals at higher risk of NTM infections, such as healthcare workers or immunocompromised patients. By measuring the release of interferon-gamma, an immune response marker, these assays can provide evidence of recent or current exposure to mycobacteria, aiding in early detection and management [47].

Histopathology, on the other hand, involves the examination of tissue biopsies obtained from affected areas. In NTM infections, histopathological examination can reveal granulomatous inflammation, a hallmark feature of mycobacterial infections. Special techniques can highlight mycobacteria within the tissues, such as acid-fast staining (using Ziehl-Neelsen or Auramine-rhodamine stains). This approach provides direct evidence of the presence of NTM organisms in the affected tissues, contributing to a definitive diagnosis and guiding treatment decisions [48].

Management and treatment of NTM infections

Antimicrobial therapy for NTM infections represents a multifaceted endeavor where the choice of antibiotics and the construction of treatment regimens are far from one-size-fits-all. These infections are caused by diverse NTM species, each with unique characteristics and susceptibilities to antimicrobial agents. Consequently, the first-line drugs, such as macrolides (e.g., azithromycin and clarithromycin), ethambutol, and rifamycins (e.g., rifampin and rifabutin), form the backbone of treatment due to their effectiveness against a broad spectrum of NTM species [49].

Macrolides are particularly crucial in NTM therapy, as they inhibit NTM organisms' growth and possess immunomodulatory properties. This dual mechanism of action can help patients with NTM infections bolster their immune responses, enhancing the overall treatment outcome. Ethambutol's role is not only to target the mycobacteria directly but also to serve as a strategic component to thwart the development of resistance to other antibiotics used concurrently [50].

Rifamycins, while not always a first-line choice, come into play in specific NTM infections like Mycobacterium kansasii or Mycobacterium abscessus. Their broad-spectrum activity makes them valuable assets in the fight against these challenging pathogens [51]. However, second-line drugs enter the scene when infections prove severe, refractory to initial treatment or involve particularly virulent NTM strains. Injectable aminoglycosides like amikacin and streptomycin are reserved for these challenging cases but come with the caveat of potential ototoxicity and nephrotoxicity, necessitating vigilant monitoring throughout the treatment course [52].

Fluoroquinolones, including moxifloxacin and ciprofloxacin, are alternative options for Mycobacterium avium complex (MAC) infections. They can be considered when other primary treatments may not suffice [53]. Linezolid, a potent antibiotic, is often reserved as a last resort for Mycobacterium abscessus infections that resist other therapeutic avenues. Its use underscores the importance of finding effective treatment strategies in challenging cases [54].

Crucially, constructing a treatment regimen for NTM infections is highly personalized. It hinges on factors such as the identified NTM species and their susceptibility to specific antibiotics. Combination therapy, involving multiple antibiotics with complementary mechanisms of action, is a common strategy. This approach enhances treatment efficacy and mitigates the risk of resistance development, a particular concern in NTM infections [25].

The duration of NTM treatment is notable for its prolonged nature, often spanning months to years. This extended timeline is incredibly accurate for pulmonary NTM infections, as complete eradication of the bacteria and prevention of relapse necessitate an extended course of treatment. Throughout this journey, clinicians must carefully balance the benefits of therapy against potential adverse effects, ensuring that the chosen antimicrobial regimen maximizes the chances of a successful outcome while minimizing harm to the patient [20].

Surgical Interventions

Surgical resection: This is a consideration in cases of localized or cavitary pulmonary NTM infections, particularly when medical therapy proves ineffective or when patients cannot tolerate the prescribed medications. This surgical approach aims to remove the infected lung tissue, which can serve as a reservoir for mycobacterial growth and persistent infection. Standard surgical procedures include lobectomy and segmentectomy, where a portion or lobe of the lung is surgically removed. These procedures can sometimes be curative, mainly when the infection is confined to a specific lung area. However, they are typically reserved for severe or recalcitrant cases due to their invasiveness [55].

Drainage procedures: When localized abscesses or empyemas (pus-filled collections) form due to NTM infection, drainage procedures become necessary to control the infection, alleviate symptoms, and prevent further complications. These drainage procedures can be performed using image-guided percutaneous techniques or traditional surgical approaches. Image-guided percutaneous drainage involves using imaging modalities such as ultrasound or CT scans to guide the placement of a needle or catheter into the abscess or collection. Surgical drainage, on the other hand, may involve open surgery to access and drain the affected area. The choice between these methods depends on the specific circumstances of the infection and the patient's overall health [56].

Challenges in Treatment

Drug resistance: NTM species can develop resistance to antimicrobial agents, posing a significant challenge in treatment. This resistance can emerge during therapy or may be intrinsic to certain NTM strains. To address this issue, regular monitoring of drug susceptibility is crucial. By conducting susceptibility testing, clinicians can detect resistance early and adjust treatment plans accordingly. Tailoring therapy to target the specific susceptibilities of the infecting NTM strain is essential to optimizing treatment effectiveness [57].

Patient adherence: NTM treatment regimens are often protracted, extending over months to years, and demand strict adherence to achieve successful outcomes. Ensuring that patients comply with their prescribed medications is paramount. Non-adherence can lead to treatment failure, infection relapse, and drug-resistant strain development. Healthcare providers play a pivotal role in educating patients about the importance of adherence and addressing any concerns or challenges that may hinder compliance. Patient support programs and regular follow-up appointments can also help maintain adherence throughout the treatment journey [58].

Treatment duration: The duration of NTM treatment is highly variable, depending on factors such as the specific NTM species involved and the site of infection. Many NTM infections require prolonged therapy to eradicate the bacteria and prevent relapse. However, the extended treatment duration can present challenges. Patients may experience drug-related side effects, affecting their quality of life and adherence to therapy. Healthcare teams must balance the need for extended treatment with managing potential adverse effects. Monitoring and managing side effects and periodically reassessing the necessity of ongoing therapy are essential components of NTM treatment [59].

NTM infections in immunocompromised individuals

HIV/AIDS Patients

Individuals grappling with HIV/AIDS confront an elevated susceptibility to NTM infections, particularly those caused by the Mycobacterium avium complex (MAC). The underlying mechanism lies in the depletion of CD4+ T cells, essential immune system components. This immunosuppression heightens vulnerability to opportunistic infections like NTM [60]. NTM infections in this population often take the form of disseminated disease, a severe manifestation characterized by systemic symptoms. These include fever, debilitating fatigue, and unexplained weight loss. Recognizing these indicators is critical for prompt intervention [61].

Management for HIV/AIDS patients with NTM infections involves a dual-pronged strategy. First and foremost, antiretroviral therapy (ART) takes precedence. ART targets HIV, suppressing viral replication, bolstering CD4+ T cell counts, and restoring immune function. Concurrently, antimicrobial treatment tailored to the specific NTM species and its susceptibility is essential to addressing the NTM infection. This multifaceted approach is crucial to managing both conditions effectively [62].

Solid Organ Transplant Recipients

Whether they have undergone lung, heart, or other transplants, solid organ transplant recipients find themselves at heightened risk for NTM infections due to the immunosuppressive medications necessary to prevent organ rejection. While these drugs are crucial for transplant success, they inevitably weaken the recipient's immune system [63].

NTM infections in transplant recipients can manifest in diverse ways, impacting various organ systems. This clinical variability necessitates high clinical suspicion for early detection [64]. Managing NTM infections in this population requires a delicate balance between controlling the underlying condition (organ transplant) and addressing the risk of infection. Physicians must continuously monitor and adjust immunosuppressive regimens to minimize vulnerability to NTM and other opportunistic infections. Antimicrobial therapy, guided by NTM species and susceptibility testing, is often necessary to combat the infection effectively [65].

Patients on Immunosuppressive Medications

Individuals receiving immunosuppressive therapies for autoimmune disorders, organ transplantation, or malignancies confront an elevated risk of NTM infections. While vital for managing their primary conditions, these medications significantly weaken the immune response, leaving patients susceptible to NTM and other opportunistic pathogens [66]. Close monitoring for NTM infections is essential in this population. Physicians must exercise careful judgment when prescribing and administering immunosuppressive drugs, considering the balance between controlling the underlying condition and minimizing the risk of infection. In some cases, adjusting or tapering immunosuppressive regimens may be necessary to reduce vulnerability to NTM infections [67].

Prevention and control of NTM infections

Infection Control Measures

Infection control measures play a pivotal role in safeguarding public health, particularly within healthcare settings where individuals may be vulnerable to infections, including those caused by non-tuberculous mycobacteria (NTM). Rigorous adherence to infection control practices is paramount to curtail the transmission of NTM and ensure patient safety. In healthcare facilities, this entails the meticulous sterilization and disinfection of medical equipment and devices, as lapses in these procedures can inadvertently lead to iatrogenic NTM infections, posing a substantial threat to patients [68].

Moreover, respiratory hygiene practices are essential in mitigating NTM transmission, particularly among individuals with respiratory NTM infections. Patients must be educated about these practices, emphasizing the importance of covering their mouth and nose when coughing or sneezing. This simple yet effective measure can significantly reduce the risk of droplet transmission, thereby protecting both patients and healthcare workers [69].

Beyond healthcare facilities, attention must also be directed towards everyday environments where NTM can lurk. Water systems in homes and healthcare facilities, including showerheads and faucets, require regular maintenance and cleaning to minimize the risk of exposure to NTM-contaminated water. This preventive step is crucial, as NTM can colonize in such systems, potentially leading to infections when individuals come into contact with contaminated water [70].

Furthermore, leisurely swimming in public pools and hot tubs should not be taken lightly concerning NTM. Proper maintenance and disinfection of these aquatic facilities are paramount to preventing NTM growth. Those who engage in such activities, particularly individuals with compromised immune systems, must be well-informed about the potential risks associated with NTM exposure. By taking appropriate precautions, such as showering before and after swimming and avoiding submersion of the head, individuals can significantly reduce their risk of NTM infection. A comprehensive approach to infection control, spanning healthcare settings and daily life, is imperative to curbing NTM transmission and safeguarding public health [71].

Public Health Strategies

Surveillance and reporting: Enhanced surveillance and reporting mechanisms are vital for monitoring NTM infections at national and global levels. Robust data collection allows for the identification of trends, the detection of potential outbreaks or clusters, and the allocation of resources for prevention and control efforts. Timely reporting can be instrumental in responding to emerging NTM threats [72].

Education and awareness: Public health campaigns should increase awareness about NTM infections, their risk factors, and preventive measures. These initiatives are crucial to educating the public and healthcare providers alike. In areas with a high prevalence of NTM infections, healthcare professionals should receive targeted education on NTM diagnostics to ensure early detection and appropriate treatment [73].

Water quality monitoring: The quality of drinking water and recreational water sources should be monitored to identify potential NTM contamination. A swift response to water quality issues is essential to preventing outbreaks and protecting public health. Regular testing and rigorous water treatment procedures can significantly reduce the risk of NTM transmission through water sources [74].

Research and guidelines: Public health agencies should invest in research to better understand NTM epidemiology and transmission patterns. This research can inform the development of evidence-based guidelines. These guidelines, tailored for both healthcare facilities and the general public, serve as valuable resources for implementing effective preventive measures. Clear guidelines guide infection control practices, diagnostic approaches, and treatment protocols [75].

Future Vaccine Development

Vaccine research: Primarily due to the vast diversity of NTM species, research into NTM vaccines is a complex endeavor. These bacteria exhibit differences in virulence, pathogenicity, and antigenic profiles, challenging the creation of a universal NTM vaccine. However, promising strides have been made in understanding specific NTM species and shared antigens. Targeted vaccines focusing on particular NTM species or common antigens shared among multiple species are being explored as a potential future preventive strategy. Such vaccines could be precious for individuals at high risk of NTM infections, such as those with compromised immune systems [76].

Immunization programs: If effective NTM vaccines are developed, public health agencies may consider incorporating them into immunization programs, especially for populations at high risk of NTM infections. Immunization programs have successfully controlled various infectious diseases, and a similar approach could prove beneficial in reducing NTM-related morbidity and mortality [75].

Advancements in vaccine technology: Advances, such as mRNA vaccines, offer exciting new possibilities for NTM vaccine development. These modern vaccine platforms provide rapid development and potential scalability advantages. Additionally, the ability to tailor vaccines to specific NTM species or antigens may become more feasible with these advanced technologies. Collaboration between researchers, government agencies, and pharmaceutical companies is crucial to advancing this field. This interdisciplinary cooperation can facilitate the development, testing, and eventual distribution of NTM vaccines, ensuring their safety and efficacy [77].

Emerging trends and research directions

Genomic Studies and NTM Evolution

Genomic diversity: Research into the genomes of different NTM species has illuminated the vast genetic diversity among these bacteria. These studies provide insights into the unique virulence factors and genetic adaptations contributing to their pathogenicity. By understanding the genomic variations within and between NTM species, scientists can better understand how these bacteria interact with their hosts and cause disease. This knowledge is pivotal for developing targeted therapeutic interventions and prevention strategies [78].

Phylogenomics: These analyses are instrumental in reconstructing the evolutionary history of NTM species and elucidating their relationships with other mycobacteria. They can identify specific genomic markers associated with virulence and drug resistance. By uncovering the genetic underpinnings of these traits, researchers can inform diagnostic and therapeutic strategies, allowing for more effective management of NTM infections [79].

Population genomics: These studies delve into the genetic diversity within NTM populations and offer insights into the epidemiology of NTM infections. This includes understanding the emergence of particular genotypes and their geographical distribution. Tracking genomic changes through these studies can help inform infection control and prevention strategies. For example, identifying the genetic basis for drug resistance can guide treatment decisions, and monitoring genomic changes can alert healthcare authorities to the emergence of drug-resistant strains, enabling a swift response to prevent outbreaks [80].

Novel Diagnostic Technologies

Advanced sequencing technologies: Next-generation sequencing (NGS) and metagenomics have emerged as powerful tools for identifying NTM species and gaining insights into their genetic profiles. These technologies enable rapid and comprehensive analysis of NTM infections, providing invaluable information for diagnosis and epidemiological studies. NGS can help detect NTM species even when conventional methods fail to do so, and metagenomics can offer a broader understanding of the microbial communities within complex clinical samples [81].

Biomarker discovery: Ongoing research efforts are dedicated to identifying specific biomarkers associated with NTM infections. Biomarkers can play a pivotal role in the early diagnosis and monitoring of treatment responses. Proteomics and metabolomics studies hold significant promise for discovering novel diagnostic markers. By analyzing the proteins and metabolites produced by NTM, researchers can uncover unique signatures that can be used for diagnostic purposes [82].

Point-of-care testing: The development of rapid, point-of-care diagnostic tests for NTM infections is a high-priority area of research. Such tests are designed to provide timely results at the patient's bedside or in resource-limited settings, eliminating the need for complex and time-consuming laboratory procedures. Point-of-care tests for NTM can significantly improve patient outcomes by enabling swift diagnosis and the initiation of appropriate treatment, reducing the risk of disease progression [83].

Advancements in Drug Development

Targeted therapies: Tailoring therapies to specific NTM species or strains is a promising approach in drug development. Understanding the genetic and metabolic vulnerabilities of NTM species enables researchers to design new antimicrobial agents that can selectively target these pathogens. By pinpointing weaknesses in NTM biology, targeted therapies aim to enhance treatment efficacy while minimizing side effects [84].

Repurposing existing drugs: Drug repurposing efforts involve identifying existing medications that may potentially combat NTM infections. This strategy leverages drugs already approved for other indications, potentially expediting the development of novel treatment regimens. Repurposed drugs can offer a faster path to clinical use since their safety profiles and pharmacokinetics are often well-established [85].

Combination therapies: Research explores the effectiveness of combination therapies to address NTM infections, particularly those associated with drug resistance. Combining multiple drugs with different mechanisms of action can reduce the risk of resistance development and improve treatment outcomes. Identifying synergistic drug combinations is a promising avenue, allowing for more effective and targeted NTM treatment regimens [86].

Multidisciplinary Approaches to NTM Research

Collaborative research networks: Scientists, clinicians, and public health experts from various disciplines are brought together to work collectively on NTM research through collaborative research networks and consortia. These partnerships pool expertise, resources, and data to accelerate research progress. By fostering cooperation and knowledge sharing, these networks enhance the exchange of ideas, methodologies, and findings, ultimately leading to a deeper understanding of NTM infections and more effective strategies for prevention and treatment [87].

Patient registries: These are established to systematically collect comprehensive clinical and epidemiological data on individuals with NTM infections. These registries are invaluable resources for researchers, allowing them to access real-world patient information. By analyzing data from these registries, researchers can conduct epidemiological studies to understand the prevalence and trends of NTM infections and gain insights into patient demographics and outcomes. Moreover, patient registries provide a valuable platform for conducting clinical trials and assessing treatment outcomes, facilitating the development of evidence-based guidelines and treatments [88].

Translational research: These initiatives bridge the gap between fundamental scientific discoveries and their clinical applications. In NTM infections, this approach is crucial for translating laboratory findings into improved patient care. Multidisciplinary teams of researchers collaborate to identify potential therapeutic targets, develop new diagnostic tools, and optimize treatment regimens. By actively translating research findings into clinical practice, translational research enhances patient outcomes and ensures that scientific discoveries have a tangible impact on healthcare [89].

## Conclusions

In conclusion, NTM infections have emerged as a significant health concern, transcending their once-underestimated prevalence. This comprehensive review has highlighted the diverse facets of NTM infections, from their epidemiology to clinical manifestations, diagnostics, treatment complexities, and preventive strategies. It has underscored the importance of early detection and management in mitigating the impact of NTM infections on affected individuals. Furthermore, the prospects in NTM research offer hope for improved understanding, faster diagnostics, and more effective treatments. With ongoing multidisciplinary collaborations and innovative approaches, we are poised to meet the challenges posed by NTM infections, ultimately enhancing the quality of care and outcomes for patients facing these complex diseases.
